# Alginate-embedded HuH-7 cells increase *MMP*-*9* and reduce *OCLN* expression in vitro

**DOI:** 10.1186/s12935-016-0370-x

**Published:** 2017-01-04

**Authors:** Virginia Andrea Angiolini, Carolina Uribe Cruz, Mónica Luján López, Laura Simon, Ursula Matte

**Affiliations:** 1 Gene Therapy Center, Hospital de Clínicas de Porto Alegre, Rua Ramiro Barcelos 2350, Porto Alegre, RS 90035-903 Brazil; 2 Post-Graduation Program in Child and Adolescent Health, Federal University of Rio Grande do Sul, Porto Alegre, Brazil; 3 Post-Graduation Program in Genetics and Molecular Biology, Federal University of Rio Grande do Sul, Porto Alegre, Brazil

**Keywords:** HuH-7, Epithelial-mesenchymal transition, Encapsulation, 3D culture

## Abstract

**Background:**

Hepatocellular carcinoma is a common cancer, ranking third in cancer-associated deaths. An important cause of cancer patients’ mortality is metastasis. At the start of metastasis progression, there is an epithelial-mesenchymal transition, characterized by matrix degradation, junction reductions and vessels formation. HuH-7 is a cell line used in research as an in vitro model for hepatocellular carcinoma. It is known that two-dimensional growth reflects tumor characteristics poorly. In contrast, three-dimensional cultures provide a better approach to the study of tumorigenic potential. The purpose of this work was to mimic a three-dimensional environment in order to assess gene expression of some epithelial-mesenchymal transition and metastasis progression markers in HuH-7 cells and compare them with traditional two-dimensional culture model.

**Methods:**

HuH-7 cells were encapsulated in sodium alginate (three-dimensional model) to be compared with cells grown in two-dimensional flasks. After 4 days in culture, gene expression of *Matrix metallopeptidase 9*, *Occludin, p65*, *Intercellular adhesion molecule 1* and *Vascular endothelial growth factor A* was analyzed by *q*PCR and cytoskeleton assessment was performed by rhodamine-phalloidin staining.

**Results:**

Differences were found in gene expression, with a high increment of *Matrix metallopeptidase 9* and *Occludin* reduction. The cytoskeleton morphology also showed differences, with a cytoplasm restricted only near the nuclei in the three-dimensional model.

**Conclusions:**

This work shows the effects of using sodium alginate capsules as a three-dimensional model to the study of HuH-7. Cells in this 3D system show key markers of epithelial-mesenchymal transition, such as *Matrix metallopeptidase 9* overexpression and *Occludin* down-regulation.

## Background

Hepatocellular carcinoma (HCC) is a common cancer, ranking sixth in the prevalence cancer list and third in cancer associated deaths [1]. The numbers of cases vary considerably worldwide and incidence is increasing over the years [2]. HCC is the fifth most common cancer in men worldwide (523,000 cases per year, 7.9% of all cancers) and the seventh most common type in women (226,000 cases per year, 6.5% of all cancers) [3].

In vitro studies using patient-derived cancer cell lines are useful as a first approach to evaluate new therapeutic targets. HuH-7 is a cell linage cells obtained from a well-differentiated hepatocellular carcinoma [4], and it is actually used in research as an in vitro model [5–7]. Although useful, there are several limitations of models using two-dimensional growth (2D), e.g., poor sensitivity to chemotherapy [8, 9], with less tumorigenicity and lower metastatic potential [10]. On the other hand, it is known that a three dimensional (3D) environment in cell culture provides a better approach to drug screening, toxicity studies and metabolic behavior [11], better mimicking the physiological contacts between cells and the mechanical properties of cells [12]. The 3D surroundings can also represent better tumor bioenergetics by generating gradients of nutrients and oxygen concentration [10].

Epithelial-mesenchymal transition (EMT) is another process not well-represented in 2D cultures and it is closely related to metastasis, an important cause of patients’ mortality caused by the spread of primary tumor cells to other tissues [13]. EMT is a process characterized by the reduction of cell to cell contact in which proteins, such as occludins (OCLN*)* (responsible for tight junction) are down-regulated. At the same time, there is an increase of matrix degradation by metalloproteinases, mainly by metalloproteinase 9 (MMP-9) [14]. These process are crucial to start the progression of metastasis [10, 14]. A following step is the formation of new blood vessels to supply the necessary nutrients for the metastatic tumor [15, 16]. In this study, different markers of EMT and metastatic progression were compared between 2D and 3D cultures using a simple and non expensive sodium alginate matrix.

## Methods

### HuH-7 cells culture

HuH-7 cells, acquired from the Cell Bank of Río de Janeiro, were grown in complete medium: DMEM (Gibco®, USA), 10% fetal bovine serum (FBS; Gibco®, USA) and 1% Streptomycin and penicillin (S/P; Gibco®, USA) and maintained in a humidified incubator at 37 °C with 5% CO_2_. The culture medium was renewed every 3 days. When confluent, cells were trypsinized (trypsin + 0.25% EDTA) (Gibco®, USA) and seeded for expansion. For the 2D model, 10^5^ cells/cm^2^ were plated and maintained for 4 days.

### Cell encapsulation

HuH-7 cells were re-suspended (3 × 10^6^ cells/mL) in 1.5% sodium alginate (Sigma Aldrich®, Germany) previously diluted in sterile complete medium (as described above). The cell suspension was passed through a syringe mounted to the encapsulation equipment (Nisco, Zurich, Switzerland) according to the following parameters: rate of 40 mL/h and air flow 4.5 L/min [17]. The suspension was dropped in 125 mM Calcium Chloride, which was replaced by complete medium after capsule formation. A total of 400 μL capsules (approximately 1.2 × 10^6^ cells) per well (n = 3) were maintained for 4 days under the same conditions mentioned above.

### Capsule depolymerization

After 4 days, capsules were collected, washed twice with 1× PBS and soaked in 1 mL of Sodium Citrate 125 Mm (Synth, Brazil) for 3 min. Subsequently, they were centrifuged at 270 g for 10 min and washed with 1× PBS. The final pellet was stored at −80 °C for later RNA extraction.

### Total RNA extraction and cDNA conversion

Total RNA extraction of cells cultured in 2D and 3D was performed using RNeasy® kit (Qiagen, Germany) according to manufacturer’s instructions. It was quantified by NanoDrop 1000 Spectophotometer (Thermo Scientific, USA) and subsequently converted to cDNA by using SuperScript™III Reverse Transcriptase kit (Invitrogen, USA) following the manufacturer’s protocol.

### Gene expression analysis

Markers characteristic of EMT and metastatic process such as, *MMP*-*9*, *OCLN*, *p65*, intercellular adhesion molecule 1 (*I*-*CAM*-*1*) and vascular endothelial growth factor (*VEGF*) were analyzed by *q*PCR using SYBR® Green (Invitrogen, USA). The list of primers, with their respective fragments sizes and annealing temperatures, are described in Table 1. Gene expression was calculated by the 2^−ΔΔCt^ value [18], using Glyceraldehyde 3-phosphate dehydrogenase (*GAPDH*) as a normalizing gene.

**Table 1:** Primers used with respective amplicon size and annealing temperatures

Gene	Primer sequence	Amplicon size (bp)	Annealing temperature (°C)
*MMP*-*9*	*f*: 5′-GCCCAGCCCACCTCCACTCCTC-3′	173	60
	*r*: 5′-TGGGCTACGTGACCTATGACAT-3′		
*OCLN*	*f*: 5′- ACAAGCGGTTTTATCCAGAGTC-3′	89	60
	*r*: 5′-GTCATCCACAGGCGAAGTTAAT-3′		
*p65*	*f*: 5′-GGGACTACGACCTGAATGCT-3′	100	62
	*r*: 5′-ATGGGATGAGAAAGGACACG-3′		
*I*-*CAM 1*	*f*: 5′-ATGCCCAGACATCTGTGTCC-3′	111	60
	*r*: 5′-GGGGTCTCATGCCCAACAA-3′		
*VEFG*	*f*: 5′-AGGGCAGAATCATCACGAAGT-3′	75	60
	*r*: 5′-AGGGTCTCGATTGGATGGCA-3′		
*GAPDH*	*f*: 5′-CCCATCACCATCTTCCAGG-	149	62
	*r*: 5′-GAGATGATGACCCTTTTGGC-3′		

### Rhodamine-phalloidin staining

A sample of encapsulated cells was fixed for 24 h with 10% formalin, washed three times with 1× PBS for 5 min and permeabilized with Triton-X-100 0.25% for 10 min. Immediately, the cytoskeleton dye rhodamine-phalloidin, diluted in a concentration of 1:100 in PBS, was applied to the capsules. It was incubated for 1 h at 4 °C in a wet and dark chamber. Finally, DAPI was dripped onto the capsules for nuclei staining and capsules were observed in *Leica* TCS SP5 (Leica, USA) confocal microscope at 40×. The same staining procedure was applied to cells grown in 2D. Photographs for 2D model were taken with an inverted Olympus IX71 (Olympus, USA) fluorescence microscope.

### Statistical analysis

Statistical analysis was performed using SPSS for Windows version 18.0. The values were expressed as mean (±standard deviation). Student’s t test was used, considering p values <0.05 as statistically significant.

## Results

**Fig. 1:**
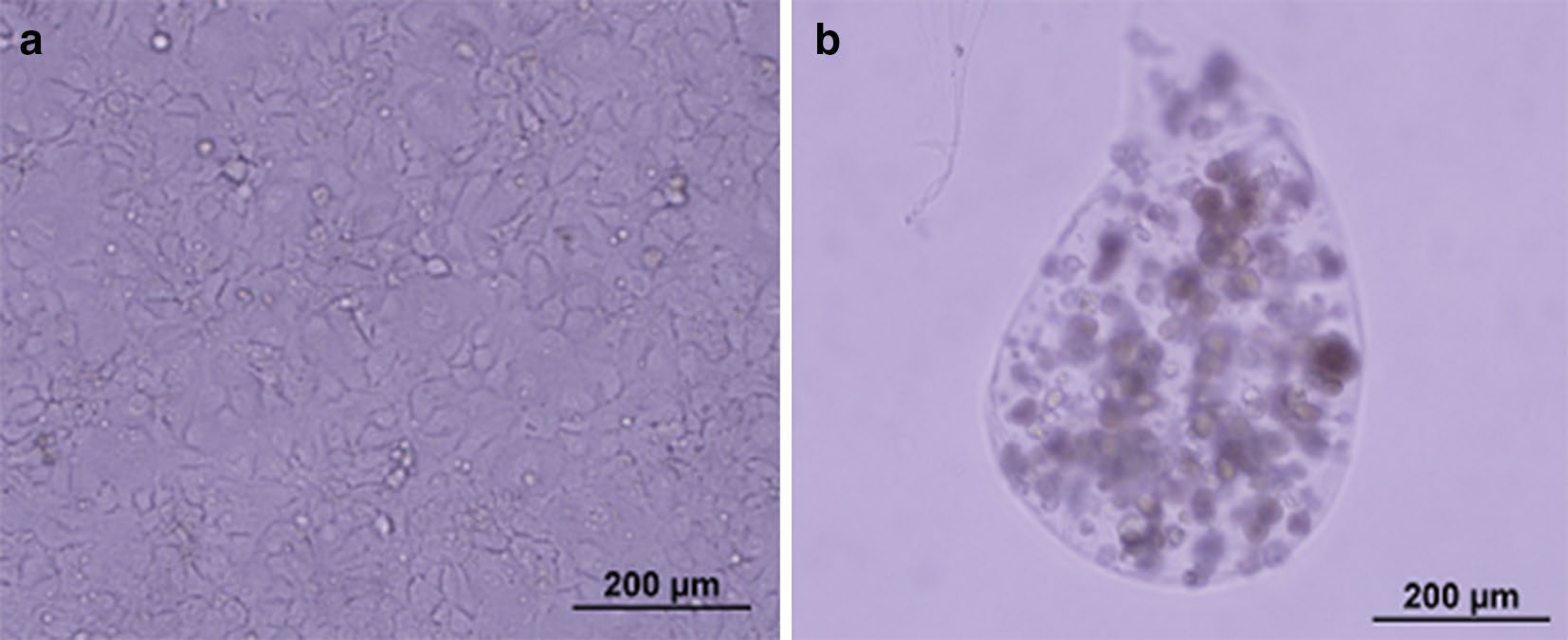
HuH-7 cells cultured in 2D flasks (**a**) or in 3D alginate capsules at day 4 (**b**). ×10

Cells previously expanded in 2D (Fig. 1a) were encapsulated in sodium alginate (Fig. 1b) and maintained for a period of 4 days. The capsules retained their integrity and morphology after the time of culture and the cells remained within them, showing that the encapsulation process is a suitable model of a 3D environment for cells.

**Fig. 2:**
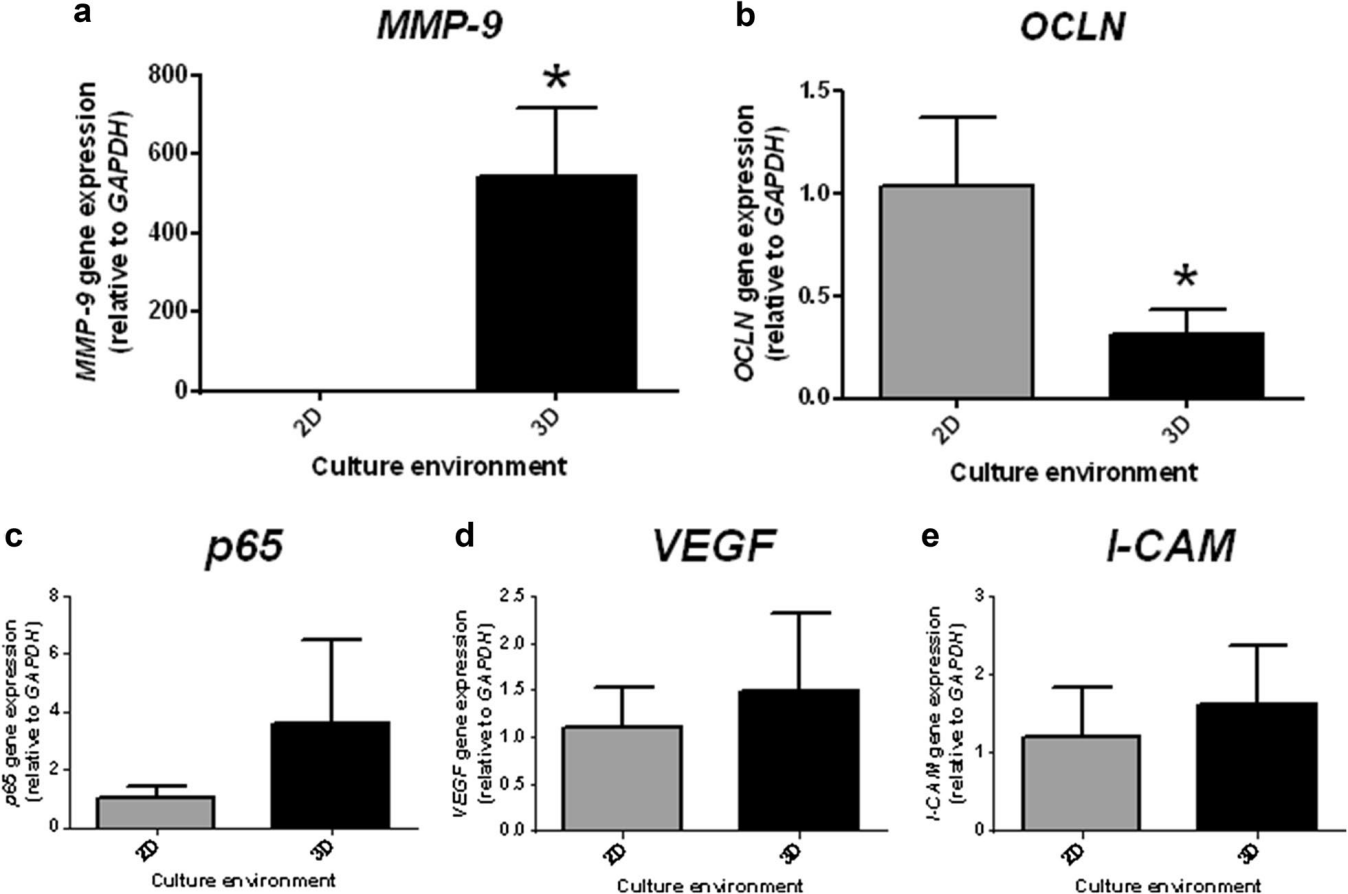
Extracellular environment provided by the alginate matrix changes the gene expression profile of HuH-7 cells towards EMT. Gene expression studies of **a** *MMP*-*9*, **b** *OCLN*, **c** *p65*, **d** *VEGF* and **e** *I*-*CAM 1*, comparing cells in 2D and 3D environment after 4 days. *p < 0.05

The HuH-7 cells encapsulated in sodium alginate matrix showed to be affected by the presence of the extracellular environment by changing their gene expression pattern. There was a marked increase in expression of the matrix remodeling protein (*MMP*-*9*) (545.03 ± 0.75 vs. 139.46 ± 0.44) (Fig. 2a). Furthermore, the expression of occludin (*OCLN*) was significantly reduced in the 3D environment (0.32 ± 0.10 vs. 1.04 ± 0.29 2D) (Fig. 2b). However, other genes that characterize the process of EMT and metastasis progression (*p65*, *I*-*CAM* and *VEGF*) showed no differences of expression between cells in the 2D or the 3D environments (Fig. 2c–e).

**Fig. 3:**
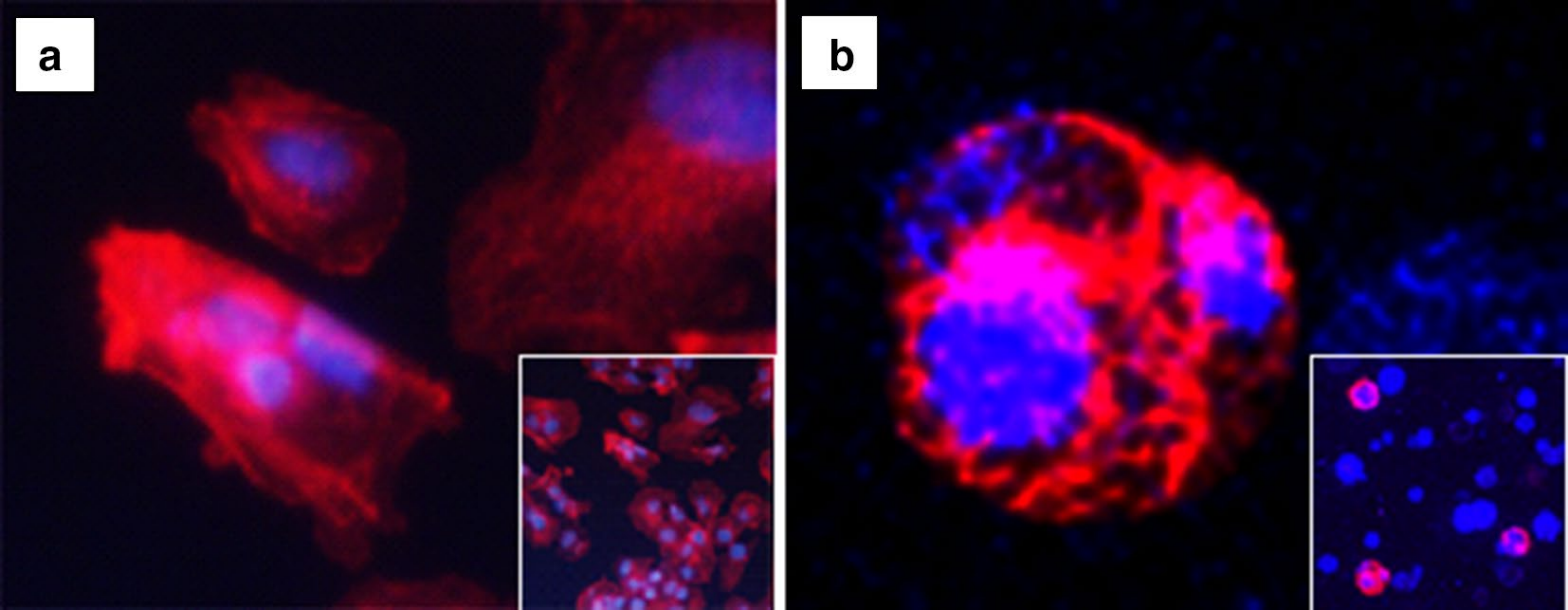
HuH-7 cells stained with rhodamine-phalloidin and DAPI in **a** 2D culture cells observed under inverted fluorescent microscopy (×20) and **b** in 3D cultured cells observed under confocal microscopy (×40)

As expected, changes in culture environment were also reflected in the cytoskeleton. Confocal microscopy with rhodamine-phalloidin staining revealed those changes (Fig. 3). Cells grown in 2D have a flattened shape and a spread cytoplasm over the plate surface (Fig. 3a). By contrast, in the 3D model, cells have a round shape with the cytoplasm being well-distributed to the nuclear periphery (Fig. 3b). Note that few cells were stained in 3D environment, probably because the dye penetrates the capsules with difficulty.

## Discussion

The use of 3D models for cell culture has many advantages over monolayer cell culture. Many different 3D models were developed for different approaches [10] and one of them is the use of alginate capsules [19]. Sodium alginate is one of the materials most commonly chosen because it form hydrogels with desirable characteristics such as lightweight, flexibility and mechanical stability [20]. Alginate encapsulation was reported to increment cell division, aid in extracellular matrix assembly and exacerbate tumor characteristics [21–23].

Our results show that HuH-7 cells change their behavior when undergo 3D culture environment, as described for other tumor cells [24]. Moreover, these changes occur in important genes for tumor progression, such as proteases and adhesion molecules.

Proteases are important to trigger cell proliferation, differentiation, matrix remodeling, vascularization and migration. All these events take place during normal development but also during malignant progression. MMP-9 is a protease widely studied because it is important to promote the whole process of cell colonization, hence proliferation, tumor invasion and metastasis [25]. According to our results, cell culture in capsules promotes a high increment in *MMP*-*9* expression. It is reasonable to think that the activation of this kind of molecule is triggered by the surrounding matrix formed by sodium alginate.

Although the three-dimensional cell culture aggregates do not always lead to the formation of a complex 3D structure, they introduce biomechanical and biophysical stimuli that promote a controlled environment promoting the assembly of “tumors tissue type” [26]. As the cells in 2D are exposed to a uniform environment with sufficient availability of nutrients and oxygen, they don’t experience the three-dimensional solid tumor environment with a gradient of chemical and critical biological signals that can benefit or harm cell growth and tumor development [8]. Hypoxia is one of the triggers of EMT, characterized by decreasing cellular junctions for the subsequent infiltration of surrounding tissues. This study observed a decrease in the expression of *OCLN,* which participates in the tight junctions between cells. This result may not due to lack of oxygen, but simply because during the process of encapsulation cells are isolated from one another, surrounded by the alginate matrix, thus unable to establish such cell contacts. However, the possibility that cells inside the capsule receive less oxygen cannot be discarded. Hypoxia-related genes and oxygen measurements must be subsequently analyzed.

However, other analyzed EMT and metastasis markers (*p65, VEGF* and *I*-*CAM*) did not differ after 4 days in 3D culture. These molecules are frequently overexpressed in 3D cultures and in cancer cells in vivo [16, 27–29]. The reasons why we failed to detect a difference in these molecules in the present study are not clear. It may be related to the length of time cells were kept in culture. An interesting analysis would be to compare results at different times, plotting temporal changes in gene expression [21]. Studies have already shown the viability of encapsulated cells for longer culture periods, up to 45 days [30]. In order to improve the model, an increment in time culture could be carried out to asses if a longer period induces different expression of other markers. It would also be important to asses different alginate concentrations to investigate the effect of this variable on EMT and metastatic progression markers in HuH-7 cells, according to studies performed for viral infections [19].

On the other hand, some limitations should be considered. Because of the small size for handling and the modifications suffering during the fixing process, it is difficult to obtain slides for histological analysis of good quality from 3D cultures. Also, as can be seen in Fig. 3, not all the encapsulated cells could be marked because of the lack of permeability of the alginate. This could hamper cytological analysis using antibodies. It would be very interesting to confirm gene expression data with protein analysis by western blot. Unfortunately, in our experience, it is very difficult to obtain good quality protein fraction from encapsulated cells, as alginate usually contaminates cell extracts, even after dissolving the beads. Nevertheless, the use of capsules as a 3D model has the advantage of decreasing the use of animals for research. Moreover, it is a cost-effective method, thus providing a useful platform for the study of cancer cells [21].

## Conclusion

Huh-7 cells change their behavior when cultured in a 3D environment, showing markers of EMT. A key marker for cell mobility and remodeling (*MMP*-*9*) was overexpressed in cells cultured in 3D, whereas *OCLN* expression was reduced. These results reinforce the use of alginate as a scaffold for 3D cultures of cancer cells.

## Abbreviations

2D: two-dimensional growth
3D: three-dimensional growth
EMT: epithelial-mesenchymal transition
FBS: fetal bovine serum
HCC: hepatocellular carcinoma
